# The impact of continuous renal replacement therapy on renal outcomes in dialysis-requiring acute kidney injury may be related to the baseline kidney function

**DOI:** 10.1186/s12882-017-0564-z

**Published:** 2017-05-03

**Authors:** Marisa Aparecida de Souza Oliveira, Thais Oliveira Claizoni dos Santos, Julio Cesar Martins Monte, Marcelo Costa Batista, Virgilio Gonçalves Pereira, Bento Fortunato Cardoso dos Santos, Oscar Fernando Pavão Santos, Marcelino de Souza Durão

**Affiliations:** 1 Nephrology Division of Hospital Israelita Albert Einstein, Avenida Albert Einstein, 627, Morumbi, São Paulo, 05652-900 Brazil; 2 Nephrology Division of Universidade Federal de São Paulo, Rua Botucatu, 740, Vila Clementino, São Paulo, 04023-062 Brazil

**Keywords:** Acute kidney injury, Dialysis, CRRT

## Abstract

**Background:**

Many controversies exist regarding the management of dialysis-requiring acute kidney injury (D-AKI). No clear evidence has shown that the choice of dialysis modality can change the survival rate or kidney function recovery of critically ill patients with D-AKI.

**Methods:**

We conducted a retrospective study investigating patients (≥16 years old) admitted to an intensive care unit with D-AKI from 1999 to 2012. We analyzed D-AKI incidence, and outcomes, as well as the most commonly used dialysis modality over time. Outcomes were based on hospital mortality, renal function recovery (estimated glomerular filtration rate-eGFR), and the need for dialysis treatment at hospital discharge.

**Results:**

In 1,493 patients with D-AKI, sepsis was the main cause of kidney injury (56.2%). The comparison between the three study periods, (1999–2003, 2004–2008, and 2009–2012) showed an increased in incidence of D-AKI (from 2.56 to 5.17%; *p* = 0.001), in the APACHE II score (from 20 to 26; *p* < 0.001), and in the use of continuous renal replacement therapy (CRRT) as initial dialysis modality choice (from 64.2 to 72.2%; *p* < 0.001). The mortality rate (53.9%) and dialysis dependence at hospital discharge (12.3%) remained unchanged over time. Individuals who recovered renal function (33.8%) showed that those who had initially undergone CRRT had a higher eGFR than those in the intermittent hemodialysis group (54.0 × 46.0 ml/min/1.73 m2, respectively; *p* = 0.014). In multivariate analysis, type of patient, sepsis-associated AKI and APACHE II score were associated to death. For each additional unit of the APACHE II score, the odds of death increased by 52%. The odds ratio of death for medical patients with sepsis-associated AKI was estimated to be 2.93 (1.81–4.75; *p* < 0.001).

**Conclusion:**

Our study showed that the incidence of D-AKI increased with illness severity, and the use of CRRT also increased over time. The improvement in renal outcomes observed in the CRRT group may be related to the better baseline kidney function, especially in the dialysis dependence patients at hospital discharge.

**Electronic supplementary material:**

The online version of this article (doi:10.1186/s12882-017-0564-z) contains supplementary material, which is available to authorized users.

## Background

Acute Kidney Injury (AKI), especially dialysis-requiring AKI (D-AKI), is associated with a poor prognosis. AKI increases the risk of developing chronic kidney disease (CKD) and death. Typically, AKI occurs while patients are hospitalized, mainly in intensive care units (ICU). These individuals usually have other comorbidities, and sepsis is a triggering factor [1–4].

Dialysis is the only specific therapy for treating AKI. The choice of dialysis modality is driven by a series of factors mainly related to the acute illness of patients, experience of nephrologists, and availability of technology and human resources. Depending on such factors, all modalities may be successfully applied to treat D-AKI [5, 6]. However, an epidemiological study has shown that the most widely used modality to treat patients with D-AKI in ICUs is continuous renal replacement therapy (CRRT) [7]. Many controversies exist regarding the management of AKI. For instance, no clear evidence has shown that the choice of dialysis modality can change the survival rate or kidney function recovery of D-AKI patients.

Therefore, we performed an epidemiological study to investigate D-AKI incidence, associated comorbidities, and outcomes for such patients, as well as the most commonly used dialysis modality in the ICU of a tertiary private hospital.

## Methods

### Type of study and length of time

A retrospective cohort study was performed in the ICU of Hospital Israelita Albert Einstein, São Paulo, Brazil, from January 1, 1999 to December 31, 2012.

The project was approved by institutional review board at Hospital Israelita Albert Einstein, under number 1715-13. Owing to the nature of the study, a waiver of consent agreement was requested. Data were kept strictly confidential.

### Inclusion criteria

Patients treated with acute dialysis (≥16 years old).

### Exclusion criteria

Patients with CKD under a regular dialysis program and kidney transplant recipients.

### Variables

Demographic, medical and laboratory (plasma creatinine and urea levels) data were collected from the hospital electronic medical records system, including the following information: age, gender, and main comorbidities such as liver disease, heart failure (HF), coronary artery disease (CAD), chronic obstructive pulmonary disease (COPD), acquired immunodeficiency syndrome (AIDS), malignant solid tumor, hematologic malignancies, solid organ transplant, and bone marrow transplant.

We analyzed the APACHE II system score [8], type of patient (medical or surgical patient), reasons for admission to the ICU, and etiology of AKI (including sepsis, ischemia due to low cardiac output, nephrotoxicity due to drugs, rhabdomyolysis, hepatorenal syndrome, and glomerulonephritis). The main sites of infection were identified in patients diagnosed with sepsis. We determined the length of stay in the hospital and ICU.

### Baseline renal function

Baseline renal function was evaluated on the basis of plasma creatinine levels by considering the lowest level within the 3-month period prior to hospitalization, when available. If such data were unavailable, we recorded the lowest plasma creatinine value during hospitalization in the period without dialysis. The simplified formula of the MDRD [9] study was used to calculate the corresponding estimated glomerular filtration rate (eGFR).

### Time and dialysis modality

No specific protocol was used to initiate the dialysis treatment or choose a certain dialysis method. Such factors were at the discretion of the nephrology team, and these factors were consistent throughout the period. Neither peritoneal dialysis nor hybrid procedures were commonly applied.

In our center, regarding the choice of initial renal replacement therapy modality, generally, unstable patients are managed with continuous therapies. These situations include: hypotensive subjects, shock, patients with multiple organ dysfunctions, use of mechanical ventilation, vasopressors, and inotropes. Patients with severe burns, multiple trauma, liver failure, brain injury, and pancreatitis are guided to CRRT as well. Less ill patients are treated with IHD.

The optimal time to start RRT in AKI remains uncertain. In addition to the usual indications such as uremia, hyperkalemia, hypervolemia, metabolic acidosis refractory to conservative measures, we usually initiate RRT in AKI in the following situations: persistently positive water balance, oliguria, severe sodium disorders, and hypercatabolism.

The first choice for vascular access placement was the right internal jugular vein. Alternatively, we used the femoral access (both sides) and the left internal jugular. Initially, we used temporary non-tunneled catheters with triple lumen. After 3 weeks of RRT and when there is no evidence of renal function improvement, we performed placement of a long-term hemodialysis catheter in internal jugular vein.

### Outcomes

Outcomes were based on hospital mortality, renal function recovery, and the need for dialysis treatment at hospital discharge. We evaluated the eGFR at hospital discharge of patients with recovering renal function.

### Follow-up period

Until hospital discharge.

### Statistical analysis

Absolute frequencies and percentages were used to describe qualitative variables. In the case of asymmetry, means and standard deviations or medians and interquartile intervals (25–75^th^) were used to describe quantitative variables. Histograms and Shapiro-Wilk normality tests were used to analyze the distribution of numerical variables.

Logistic regression models for simple and multiple regressions were used to analyze the factors associated with death among patients with AKI. After selecting the variables, interactions were investigated. Only the final multiple model obtained is shown.

The SPSS program (Version 17.0. Chicago: SPSS Inc.) was used to perform statistical analyses, and the significance level cutoff was 95%.

## Results

Within a 14-year period, we identified 1,493 patients with D-AKI (Table 1). Most were male (65.0%) and white (92.5%), and their average age was 63 ± 18 years. The main comorbidities were diabetes (36.2%), systemic arterial hypertension (36.0%), and cardiovascular disease (36.0%). A total of 526 individuals had been submitted to solid organ transplants (35.2%), mainly liver transplants. We found 839 cases of AKI related to sepsis (56.2%). Others causes of AKI were low cardiac output (*N* = 270, 18.1%), drug-induced nephrotoxicity (*N* = 186, 12.5%), hepatorenal syndrome (*N* = 81, 5.4%), rhabdomyolysis (*N* = 74, 5.0%), glomerulonephritis (*N* = 23, 1.5%), and unknown (*N* = 20, 1.3%). The most common types of infection were pulmonary (43.7%) and abdominal (37.3%).

**Table 1:** Description of the main clinical and demographic data

Variables	(*N* = 1,493)	%
Gender (male) *N* (%)	970	65.0%
Age (years) mean (SD)	63	18
Race N (%)		
White	1381	92.5%
Comorbidities *N* (%)		
DM	541	36.2%
AH	537	36.0%
HD	528	35.4%
COPD	153	10.2%
Malignant solid tumor	200	13.4%
Hematologic malignances and BMT	107	7.2%
Cirrhosis/other liver diseases	100	6.7%
Solid organ transplant	526	35.2%
Liver	509	34.1%
Heart	14	0.9%
Lung	2	0.1%
Multivisceral	1	0.1%
Type of patient *N* (%)		
Medical	1094	73.3%
Surgical	399	26.7%
Reason for admission to ICU N (%)		
Infection	577	38.6%
Cardiovascular disease	399	26.7%
Pancreatitis	58	3.9%
Bleeding	48	3.2%
Intoxication	12	0.8%
Surgical urgency	339	22.7%
Elective surgery	19	1.3%
Trauma	33	2.2%
Major burn	8	0.5%
AKI etiology *N* (%)		
Sepsis	839	56.2%
Low cardiac output	270	18.1%
RRT modality *N* (%)		
IHD	501	33.6%
CVVHDF	992	66.4%
Death *N* (%)	805	53.9%
Baseline creatinine (mg/dl) median (IQR)	0.9	(0.7–1.5)
eGFR–ml/min/1.73 m^2^ median (IQR)	84	(49–106)
APACHE II median (IQR)	23	(18–30)
ICU length of stay (days) - median (IQR)	12	(6–24)
Hospital length of stay (days) - median (IQR)	30	(15–52)

*DM* diabetes mellitus, *AH* arterial hypertension, *HD* heart diseases, *COPD* chronic obstructive pulmonary disease, *BMT* bone marrow transplantation, *ICU* intensive care unit, *RRT* renal replacement therapy, *IHD* intermittent hemodialysis, *CVVHDF* continuous venovenoushemodiafiltration, *eGFR* estimated glomerular filtration rate, *APACHE* Acute Physiology And Chronic Health Evaluation, *SD* standard deviation, *IQR* interquartile range

The APACHE II score was 23 (18–30), and the basal eGFR was 84 (49–106) ml/min/1.73 m^2^.

The most commonly used dialysis modality was continuous dialysis (66.4%), consisting exclusively of venovenous hemodiafiltration (CVVHDF); the second most common was intermittent hemodialysis (IHD) (33.6%). Of all patients who initially performed IHD, 136 individuals (27%) had to undergo CVVHDF due to medical condition worsening during the course of the disease. Of those who first performed CVVDHF, 526 subjects (53%) were de-escalated for IHD due to illness improvement.

The overall death rate was 53.9%. The ICU and hospital lengths of stay were 12 (6–24) and 30 (15–52) days, respectively. In addition, 12.3% patients required dialysis at hospital discharge. The remaining patients (33.8%) recovered at least partial renal function.

### Comparison of the APACHE II scores, on the basis of dialysis modality

The APACHE score was assessed according to initial dialysis method. The APACHE median was 19 (16–25) for patients who had undergone IHD and 26 (20–30) for those who had undergone CVVHDF (*p* < 0.001).

### Comparison of the death rates, on the basis of dialysis modality

The death rate was 64.2% for patients who had undergone CVVHDF and 33.5% for those who had undergone IHD (*p* < 0.001). However, as reported above, on the basis of APACHE II scores, the severity of illness was greater for individuals who had initially undergone CVVHDF.

### Comparison of dialysis dependence at hospital discharge, on the basis of dialysis modality

Patients who had initially undergone IHD showed a greater dependence on dialysis support at hospital discharge than those who had initially undergone CVVHDF (33.6 vs. 20.3%, *p* < 0.001). However, significant differences were observed relative to the baseline eGFR among individuals in the two groups. Patients who had undergone IHD had a lower baseline eGFR than those who had undergone CVVHDF (43.3 and 85.1 ml/min/1.73 m^2^, respectively, *p* < 0.001) (Table 2).

**Table 2:** Renal function and outcomes between the IHD and CVVHDF groups

	IHD (501)	CVVHDF (992)	*P* value
APACHE score	19 (16–25)	26 (20–30)	<0.001
Creatinine baseline (mg/dL)^a^	1.05 (0.8–1.9)	0.9 (0.7–1.2)	<0.001
eGFR baseline (mL/min/1.73 m^2^)^a^	69.5 (34.1–100.9)	87.6 (51.1–109.2)	<0.001
Creatinine in the beginning of RRT (mg/dl)	3.7 (3.1–4.5)	3.6 (3.1–4.1)	<0.001
Urea in the beginning of RRT (mg/dL)	139 (106–176)	140 (107–174)	0.596
Death	168 (33.5%)	637 (64.2%)	<0.001
Patients who remained dependent on dialysis at hospital discharge^b^	112 (33.6%)	72 (20.3%)	<0.001
eGFR baseline (mL/min/1.73 m^2^) in dialysis dependence patients	43.3 (26.9–86.0)	85.1 (49.4–99.5)	<0.001
Patients who recovered renal function^b^	221 (66.4%)	283 (79.7%)	<0.001
eGFR baseline (mL/min/1.73 m^2^) in renal recovery patients	79.3 (42.3–107.2)	87.8 (51,7–110.5)	0.101
Creatinine discharge (mg/dL) in renal recovery patients	1.6 (1.1–1.9)	1.3 (1.0–1.8)	0.014
eGFR discharge (mL/min/1,73 m^2^) in renal recovery patients	46.0 (34.6–68.8)	54.0 (38.7–72.2)	0.011

Values expressed as median (interquartile range) or number (percentage)

*IHD* intermittent haemodialysis, *CVVHDF* continuous venovenous hemodiafiltration, *APACHE* Acute Physiology And Chronic Health Evaluation, *eGFR* estimated glomerular filtration rate, *RRT* renal replacement therapy

^a^488 patients in de IHD group and 960 patients in CVVHDF group

^b^% values calculated from the number of survivors

### Comparison of the eGFR at hospital discharge (individuals who recovered renal function), on the basis of dialysis modality

A comparison of the eGFR at discharge and the initial dialysis modality chosen was performed. The median eGFR was 46.0 (35–68) ml/min/1.73 m^2^ for patients who underwent IHD and 54.0 (39–72) ml/min/1.73 m^2^ for those who underwent CVVHDF (*p* = 0.014).

In the group of survivors who recovered renal function, the baseline median eGFR was 79.3 (42–107) ml/min/1.73 m^2^ for patients who underwent IHD and 87.8 (52–110) ml/min/1.73 m^2^ for those who underwent CVVHDF (*p* = 0.101) (Table 2).

### Comparison of the three periods: 1999–2003, 2004–2008 and 2009–2012

To evaluate the changes in multiple variables over time, the total study time was divided into three periods: 1999 to 2003 (5 years), 2004 to 2008 (5 years) and 2009 to 2012 (4 years). The numbers of patients hospitalized in the ICU during these three periods were 10,565, 12,312 and 12,251, respectively.

These three periods are compared in Table 3. Significant changes were observed in the percentage of patients with liver disease who were submitted to liver transplantation, which increased from 19.2% during the first period to 41.2% during the third period (*p* < 0.001).

**Table 3:** Comparison of the three periods (time-stratified data)

	Evaluated periods						*P*
	1999–2003		2004–2008		2009–2012		
AKI N (incidence-%)	271 (2.56%)		588 (4.77%)		634 (5.17%)		0.001
Liver disease and liver transplant *N* (%)							
Cirrhosis/other liver diseases	21	7.70%	27	4.60%	52	8.20%	<0.001
Liver transplant	52	19.20%	197	33.50%	261	41.20%	
Heart disease *N* (%)	94	34.70%	228	38.80%	206	32.50%	0.069
Malignant solid tumor *N* (%)	42	15.50%	78	13.30%	80	12.60%	0.504
Solid organ transplant *N* (%)	52	19.20%	200	34.00%	274	43.20%	<0.001
Hematologic malignances and BMT *N* (%)	19	7.00%	46	7.80%	42	6.60%	0.715
Sepsis-AKI *N* (%)	148	54.60%	329	56.00%	362	57.10%	0.779
RRT modality *N* (%)							
Hemodialysis	97	35.80%	228	38.80%	176	27.80%	<0.001
Continuous dialysis	174	64.20%	360	61.20%	458	72.20%	
Outcomes *N* (%)							
Death	158	58.30%	312	53.10%	335	52.80%	0.352
Dialysis dependence	26	9.60%	81	13.80%	77	12.10%	
Recovery of renal function	87	32.10%	195	33.20%	222	35.00%	
Vasopressors *N* (%)	257	94.80%	558	94.90%	593	93.50%	0.541
Mechanical ventilation *N* (%)	231	85.20%	502	85.40%	544	85.80%	0.966
APACHE II score median (IQR)	20	(19–22)	25	(18–29)	26	(18–31)	<0.001
Age (years) mean (SD)	64	18	65	18	61	18	<0.001

*AKI* acute kidney injury, *BMT* bone marrow transplantation, *RRT* renal replacement therapy, *APACHE* Acute Physiology And Chronic Health Evaluation, *SD* standard deviation, *IQR*, interquartile range

A significant change was also found regarding the choice of initial dialysis method during the time periods. CVVHDF selection increased from 64.2% during the first period to 72.2% during the third period (*p* < 0.001).

A significant change in the APACHE II score was also observed. The median score was 20 during the first period, 25 during the second period and 26 during the third period (*p* < 0.001). No significant changes were found in the percentage of sepsis-induced AKI cases, use of vasopressors, use of mechanical ventilation or patient outcomes.

### Mortality risk factors

By univariate analysis, heart disease (1,38 [1,12-1,71; *p* = 0,003]), hematologic malignancy and bone marrow transplantation (2,21 [1,44-3,40; *p* < 0.001]), cirrhosis and liver transplantation (2,61 [1,66–4,11; *p* < 0.001]), solid organ malignancy (1,30 [0,96-1,76; *p* = 0.089]), surgical/medical patients (0,42 [0,33 - 0,53; *p* < 0.001]), sepsis-associated AKI (2,06 [1,68-2,54; *p* < 0.001]), CRRT as first choice dialysis modality (3,56 [2,84-4,46; *p* < 0.001]), use of vasopressor (14,58 [6,68-31,81; *p* < 0.001]), use of mechanical ventilation (7,57 [5,20-11,01; *p* < 0.001]), APACHE II score (1,54 [1,48-1,60; *p* < 0.001]) and baseline eGFR (1,00 [1,00– 1,01; *p* = 0.067]) correlated with death.

In the multivariate analysis, variables that remained significant in the model were selected, including type of patient, sepsis-associated AKI and APACHE II score. We observed a significant interaction between the patient type and etiology of AKI; i.e., the effect of patient type on the risk of death depends on the etiology of AKI. From this finding, we constructed a variable according to the combination of the patient type and the etiology of AKI, with the following categories: medical patient with non-sepsis-associated AKI, medical patient with sepsis-associated AKI, surgical patient with non-sepsis-associated AKI and surgical patient with sepsis-associated AKI. The results of the final adjusted model are shown in Table 4.

**Table 4:** Multivariate analysis of the factors associated with death

	Odds ratio (95% CI)	*p*
Type of patient and cause of AKI		
Medical patient with non-septic-AKI/	2.54 (1.50–4.32)	0.001
Surgical patient with non-septic-AKI		
Medical patient with septic-AKI/	2.93 (1.81–4.75)	<0.001
Surgical patient with non-septic-AKI		
Surgical patient with septic-AKI/	2.50 (1.27–4.92)	0.008
Surgical patient with non-septic-AKI		
APACHE II score	1.52 (1.46–1.58)	<0.001

*AKI* acute kidney injury, Acute Physiology And Chronic Health Evaluation, *CI* confidence interval

For each additional unit of the APACHE II score, the odds of death increased by 52%.

The odds ratio of death for medical patients with sepsis-associated AKI was estimated to be 2.93 [1.81–4.75; *p* < 0.001].

## Discussion

Our cohort showed an increase in D-AKI rates in the ICU over time. Observational studies have also indicated that the overall incidence of AKI has increased during recent decades [10, 11]. The incidence of AKI requiring dialysis has also increased [12, 13]. Recent data have shown that most individuals develop AKI while being hospitalized in the ICU. In this scenario, the need for dialysis ranges from 5 to 10% [7, 14]. Similar rates were found in our sample.

Although we did not observe an increase in the use of vasopressors or mechanical ventilation at the beginning of the dialysis therapy, a temporal analysis showed an increase in the severity of illness, a larger number of patients submitted to solid organ transplants, mainly liver transplants, and an increase in patients with cirrhosis and other liver diseases. The number of solid organ transplants has been increasing worldwide [15]. Patients often experience medical conditions that worsen during the perioperative period (e.g., hypotension, shock, and bleeding). Because of the use of immunosuppressive medications, they are subject to infections, complications from drug interactions, and cardiovascular disorders, all of which ultimately lead to ICU admissions.

Liver transplant patients are susceptible to AKI development mainly because of the effects of liver disease on renal function, but also because of the reasons mentioned above [16]. Several studies have shown that some liver transplant recipients have hepatorenal syndrome, and others use nephrotoxic drugs as antibiotics and contrast agents during the pre-transplant period. Hypotension and the use of vasopressors, graft dysfunction, the need for surgical intervention, transfusion of blood products and bacterial infections are some of the most common factors associated with the development of AKI during the liver post-transplant period [17, 18].

This study showed that sepsis is a leading cause of AKI over time (greater than 50% of the cases). Despite the seemingly decreasing rate of mortality from sepsis, some studies have shown that the rates of sepsis or septic shock have been increasing at an annual rate of 8 to 13% [19, 20]. In this context, data have also indicated that sepsis is the main factor associated with the development of AKI, especially in the ICU [21, 22]. A clear association has also been found between the severity of illness and the stage of AKI [23–25]. In our sample, we noted an increased APACHE II score over time concomitant with the increased incidence of D-AKI.

The initial dialysis modality most often used was CVVHDF, which was also observed to increase over the three periods studied. Epidemiological data have shown that continuous modalities are more widely used in the treatment of patients with AKI in the ICU. In some regions, CRRT is used exclusively [7]. Recent, larger trials have shown that sicker patients who are hemodynamically unstable tend to receive continuous or hybrid therapies [22, 26]. The theoretical advantage of this type of procedure is that it allows for greater removal of fluids while maintaining hemodynamic stability. The constant removal of solutes thus avoids sudden changes in plasma osmolality, especially in individuals with intracranial hypertension. Continuous modalities provide better adjustment of metabolic acidosis and metabolic control for hypercatabolic individuals, and induce possible immunomodulatory effects [27–29].

Despite advances in the management of patients with AKI, most studies have shown persistently high mortality rates. Others have indicated a slight decrease in mortality [30]. In our study, regardless of the increase in the APACHE II score, the mortality rate remained unchanged over time. Individuals who had initially undergone CVVHDF had a higher mortality rate than those who had undergone IHD. However, patients in the CVVHDF group had a greater APACHE II score at ICU admission, which increases the difficulty of assessing the two groups. Most studies that have compared continuous therapies with IHD have shown no evident benefit with regard to survival rates [31]. Larger trials have evaluated the dialysis dose for patients with AKI in the ICU. In these studies, the most severe patients were directed to CRRT as the first choice of treatment, which appears to be a universal practice [22, 26]. Recently, hybrid therapies have emerged as an alternative to treat such individuals and appear to have the same advantages as CRRT [32].

Another important aspect regarding outcomes is dialysis dependence at hospital discharge and in the long-term. Some observational studies have suggested that dialysis therapies may influence the recovery of renal function after an episode of AKI. Thus, continuous therapies seem to decrease the need for dialysis compared with conventional hemodialysis [33, 34]. In our cohort, we observed that the subjects who had initially undergone IHD had a higher rate of dialysis dependence at hospital discharge than those who underwent CVVHDF first. However, we noted that the individuals in the IHD group had a lower baseline eGFR than those in the CVVHDF group. Because the presence of CKD is one of the main risk factors for lasting kidney impairment after an AKI episode, it was impossible to compare these groups.

A simple analysis of individuals who survived and recovered renal function showed that those who had initially undergone CVVHDF had a higher GFR than those in the IHD group at time of hospital discharge. No significant difference was observed in the baseline eGFR between the two groups. Theoretically, continuous therapy may preserve kidney function, thereby maintaining the hemodynamic status and reducing hypotension episodes, especially during fluid withdrawal. However, eGFR at hospital discharge may overestimate renal function of these individuals in both groups (CVVHDF and IHD) because of loss of muscle mass that occurs during the course of a serious illness [35].

In the multivariate analysis, the APACHE II score and sepsis-associated AKI in medical patients were independent risk factors related to death. Bagshaw and cols. have shown that sepsis-induced AKI occurs more frequently in medical patients with higher severity illness scores on ICU admission. Such individuals have longer hospital stays and exhibit higher mortality rates than those of patients with AKI not associated with sepsis [36].

Our study has some limitations. This is an observational, single-center study, and the comparative analysis between groups was subject to bias. No specific protocol was used for the choice of the first dialysis modality. The decision was made by the nephrology team, although the team remained constant throughout the study period. Our center performs only CVVHDF and IHD. Other modalities, such as hybrid therapies and peritoneal dialysis, were not addressed. The timing of RRT initiation and dialysis dose were not evaluated. The follow-up period was restricted to the time of hospital discharge, and some AKI complications may arise in the medium- and long-term periods.

Often, unstable patients do not tolerate IHD and are guided to CRRT. There is also a transition between therapies during the evolution of the disease, which makes it difficult to perform randomized clinical studies comparing the dialysis modalities.

## Conclusions

In conclusion, our study showed that the incidence of D-AKI increased with illness severity, and the use of CRRT also increased. Sepsis was the main cause of AKI. The mortality rate and need for dialysis after an episode of AKI remained stable over time. The improvement in renal outcomes observed in the CRRT group may be related to the better baseline kidney function, especially in the dialysis dependence patients at hospital discharge. Medical patients, sepsis-associated AKI and higher APACHE II scores were the independent risk factors associated with death. Strategies to reduce or enable a specific treatment for sepsis-associated AKI should be established. Despite the difficulties, randomized clinical trials comparing continuous and intermittent therapies are needed to evaluate the recovery of renal function as a primary outcome.

## Additional file

Additional file 1: Data generated. (XLS 520 kb)

## Abbreviations

AIDS: Acquired immunodeficiency syndrome
AKI: Acute kidney injury
CAD: Coronary artery disease
CKD: Chronic kidney disease
COPD: Chronic obstructive pulmonary disease
CRRT: Continuous renal replacement therapy
CVVHDF: Continuous venovenous hemodiafiltration
D-AKI: Dialysis-requiring AKI
EGFR: Estimated glomerular filtration rate
HF: Heart failure
ICU: Intensive care unit
IHD: Intermittend hemodialysis
